# Nurses’ knowledge about palliative care and attitude towards end- of-life care in public hospitals in Wollega zones: A multicenter cross-sectional study

**DOI:** 10.1371/journal.pone.0238357

**Published:** 2020-10-07

**Authors:** Werku Etafa, Bizuneh Wakuma, Getahun Fetensa, Reta Tsegaye, Eba Abdisa, Adugna Oluma, Tadesse Tolossa, Diriba Mulisa, Tagay Takele

**Affiliations:** 1 School of Nursing and Midwifery, Institute of Health Science, Wollega University, Nekemte, Ethiopia; 2 Department of Public Health, Institute of Health Science, Wollega University, Nekemte, Ethiopia; 3 Department of Mathematics, College of Natural and Computational Science, Wollega University, Nekemte, Ethiopia; University of California San Diego, UNITED STATES

## Abstract

**Background:**

Palliative care is nowadays essential in nursing care, due to the increasing number of patients who require attention in the final stages of their life. Lack of knowledge of and negative attitude palliative care among nurses is one of the most common barriers to quality palliative care. This study, therefore, aimed to assess nurses’ knowledge about palliative care and attitude toward end-of-life care in public hospitals in Wollega zones, Ethiopia.

**Methods:**

A multicenter institutional-based cross-sectional study design was employed to collect data from 372 nurses working in public hospitals in Wollega zones from October 02–22, 2019. A self-administered questionnaire with three different parts: Demographic characteristics of nurses, the Palliative Care Quiz for Nursing (PCQN), and the Frommelt Attitudes Towards Care of the Dying (FATCOD). SPSS version 21 was used for analysis used for data analysis. The binary logistic regression test was used for analysis at p < 0.05.

**Findings:**

Our final sample size was 422 nurses (response rate = 88%). With the mean total PCQN scores (9.34), the majority of them showed an inadequate level of knowledge about palliative care. The mean total FATCOD scores (79.58) displayed a positive attitude toward end-of-life care, with 52% of respondents eager to care for a dying person and their family. Nurses who had PC service experience [AOR = 1.94 CI (1.10–3.42), p = 0.02] and had ever attended training/lecture on PC [AOR = 1.87 CI (1.01–3.46), p = 0.04] were independently associated with nurses’ knowledge about PC. Similarly, nurses who had no PC service experience [AOR = 0.41, CI (0.21–0.79), p = 0.008], who read articles/brochures about PC [AOR = 1.94, CI (1.11–3.39), p = 0.01] and had provided care for a smaller number of terminally ill patients [AOR = 1.74, CI (1.01–2.97), p = 0.04] were significantly associated with nurses’ attitude towards end-of-life care.

**Conclusion:**

The study highlighted that nurses’ knowledge about palliative care is inadequate, and showed a less favorable attitude toward end-of-life care. The findings also provide evidence for greater attentions and resources should be directed towards educating and supporting nurses caring for patients with palliative care needs in Wollega Zones.

## Introduction

The World Health Organization (WHO) defined palliative care (PC) as “an approach that improves the quality of life of patients and their families facing the problem associated with a life-threatening illness, through the prevention and relief of suffering through early identification and impeccable assessment and treatment of pain and other problems, physical, psychosocial and spiritual” [1].

More recently, palliative care has seen its goal expanded from a view of its intense care of a patient who is close to death to patients who may live for many years with end-stage organ failure or cancer and aimed to relieve the pain, suffering [2]. Access to palliative care has been recognized as the basic human right that should be provided for all human beings regardless of their disease type, income and age [3, 4].

The rising of chronic non-communicable disease and increasing population aging are contributing for the global need of palliative care [5]. However, in most of countries there is still a large unmet need for palliative care service [6]. According to WHO’s 2014 global atlas of palliative care reported, 40–60% of estimated deaths needed palliative care. Cardiovascular diseases (38.5%) and cancer (34%) were among the commonest diseases of adults in need of palliative care. The majority (98%) of children needing PC live in low- and middle-income countries with almost 50% of them living in Africa [4].

The provision of hospital palliative care reduces costs by shortening the length of hospital stay [7]. The World Health Organization has founded a public health strategy (PHS) as the effective approach to incorporate palliative care in to the country’s health care system [8]. This strategy covers appropriate policies, adequate drug availability, education of policy makers, health care workers, and the public; and implementation of palliative care services at all levels throughout the society [8]. Palliative care services are more commonly takes place in hospital settings [9].

Nurses allocate more of their time with patients and families than other disciplines [10]. A 2016 systematic review reported that nurses as the most common interventionists working in teams or as an individual practitioner as of studies [11]. In the end-stage of life threatening diseases like cancer, treatment of pain and other symptoms must be considered to preserve the quality of life [12]. End-of-life care is one of the routine activities of nurses [13]. The palliative care service is successfully delivered through the combined effect of knowledge, attitudes, beliefs, and experiences of health care providers [14, 15]. Nurses’ feeling of poor preparation and stress contributes nurses to the exacerbation of negative attitudes toward death and caring for the dying that may further impact on the standard of care [16].

A literature review indicated that several studies assessed nurses’ knowledge about palliative care using the Palliative Care Quiz for Nursing (PCQN). Ronaldson et al. (2008) found that a mean score of 11.7 and 5.8 for registered and assistant nurses, respectively [17]. Brazil et al. (2012) using PCQN reported the average correct score ranged from 52.50% to 63.41% among Canadian palliative care nurses [18]. Additionally, by utilizing a similar study tool, Kassa et al. (2014) displayed the majority (69.5%) of nurses working in private and public hospitals in Addis Ababa, had poor knowledge score (less than the mean score) [19]. Abudari et al. (2015) also displayed a knowledge deficit (mean score 9.06, less than the average among nurses working in tertiary care facilities and regional cancer centers [20].

Similarly, some studies [19–21] displayed a positive /favorable attitude about palliative care using the Frommelt Attitude Toward Care of the Dying Scale (FATCOD) instrument. In contrast, few studies showed that Kim and Hwang (2013) reported unfavorable attitudes among nurses toward palliative care for those with heart failure patients [22]. A structured cross-sectional study undertaken among 553 nurses working at a teaching hospital in Malaysia indicated unfavorable attitude towards end-of-life care [23].

Studies [19, 20, 22, 24, 25] showed that the absence of palliative care curriculum content, training in palliative care, years of nursing experience, institution, individuals’ level of education, and work unit significantly affects nurses’ knowledge to practice PC and/or their attitude toward palliative care. Integrating theoretical and practical training on palliative care improved students' knowledge and creates favorable attitudes toward death, dying, and end-of-life care [26]. Another Study showed that education on clinical experience could change healthcare professionals’ attitude toward ends-of-life care [27].

There is a lack of palliative care units in Ethiopia, particularly in Wollega public hospitals. Patients who are seriously ill with a life-threatening illness receive care in general hospitals. All nurses currently working in public hospitals in Wollega zones are eligible to deal with the challenges of palliative care. To our knowledge, no study evaluated nurses’ knowledge about PC and attitude toward end-of-life care among nurses working in public hospitals in Wollega zones. The findings from this study will provide important information for the gap in the area which will help the concerned bodies to tackle this public health problem. This study was aimed to evaluate the nurses’ knowledge about palliative care and attitude towards end-of-life care among nurses working in public hospitals in Wollega zones.

## Methods

### Study design, area and period

A facility-based multicenter cross-sectional study design was employed to collect data from nurses working in public hospitals in Wollega zones from October 02–22, 2019. There are 15 public hospitals in Wollega zones. Nine randomly selected public hospitals were involved in this study. These hospitals were: Wollega University Referral, Nekemte Specialized, Gimbi, Nedjo, Shambu, Sire, Guduru, Jimma Arjo and Dambi-Dollo hospitals. These hospitals had 110, 95, 73, 57, 70, 54, 53, 56 and 68 nurses, respectively. In general, 568 nurses were found in the selected public hospitals.

### Study subjects

All randomly selected (using the lottery method) and consented to participate, and working in selected hospitals of Wollega zones participated in the study. Nurses who are on the annual leave during the data collection period, not employed as full-time nurse, and who had experienced less than 1 year were excluded from the study.

### Sample size and sampling techniques

The required sample size was determined by using a single population proportion formula. The prevalence of the nurse’s knowledge and attitude regarding palliative care in the study area is unknown. Hence, we took the proportion to be 50% (p = 0.5) to have a larger sample size. By using a 5% marginal error, we obtained 384. Finally, adding a non-response rate of 10%, the final sample size was 422. This study was conducted among nurses working in nine public hospitals located in Wollega zones. A Simple random sampling technique (lottery method) was employed to select the study participants by using lists of nursing staff as a sampling frame. Then, the number of participants in each selected hospitals were determined using the population proportionate sampling to each randomly selected public hospitals. Accordingly, proportion allocation to population size for each hospital was 73, 63, 47, 48, 35, 37, 36, 38 and 45 to Wollega University Referral, Nekemte Specialized, Shambu, Gimbi, Guduru, Jimma Arjo, Sire, Nedjo and Dambi-Dollo hospitals, respectively.

### Study instrument

A translated version of the PCQN and FATCOD were utilized to assess nurses’ knowledge about palliative care and attitude towards end-of-life care, respectively. They have been used throughout the world. A self-administered questionnaire used for data collection contained three different parts (Additional file1). The first part was demographic characteristics of nurses such as level of hospitals, age, level of education, work experience, grade point average, past palliative care attendance of training or lecture, duration of training/lecture attendance, work unit, read articles about PC, work status, whether studying currently.

Part two of the data collection tool was adopted from Palliative Care Quiz for Nursing (PCQN) [28]. This scale indicated high content validity, and reasonable reliability (test–re-test = 0.56 and Kuder-Richardson 20 = 0.78). The validity and reliability of the PCQN have been checked in previous research studies [21, 23]. These authors found an acceptable validity and reliability for PCQN.

The PCQN contains 20 questions, which can be grouped into three categories and aggregated to yield a total score. Total scores can range from 0 (the lowest level of knowledge) to 20 (the highest level of knowledge). It is categorized in three subscales including (1) philosophy and principles of palliative care (4 items), (2) management of pain and symptoms (13 items) and, (3) psychosocial and spiritual care (3 items). The options for answers are true, false, and “I do not know.” The final answers are coded 1 = correct, 0 = incorrect and I do not know.

The third part of the data collection tool was adopted from the Frommelt Attitudes Towards Care of the Dying (FATCOD) used to assess nurses’ attitudes towards the care of dying patients and their families, which consists of 30 items [29]. However, after a pilot study was conducted, 24 items were utilized in the final study tool that scored on a Likert scales ranging from strongly disagree to strongly agree: 1 = strongly disagree, 2 = disagree, 3 = unsure, 4 = agree, and 5 = strongly agree. The possible overall scores range from 24 to 120, with higher scores reflecting more positive attitudes. The scale contains 12 negative and 12 positive statements, with reversed scores assigned to negative statements.

The content validity of the scale for both PCQN and the FATCOD items were assessed by five members from the school of nursing and midwifery holding a minimum of assistant professor rank and experienced researchers ensured the cultural and linguistic validity of instrument. They agreed that PCQN is a linguistic, culturally, and religiously appropriate questionnaire to be used in the research context. However, six items from the FATCOD scale were decided to be removed from the study because of their difficulty to understand and increase bulkiness of the questionnaires. The internal consistency reliability among 5% of the sample (n = 21) for this study was 0.75 for PCQN and 0.77 for FATCOD scales which showed acceptable reliability.

For the translation of the questionnaire (both the PCQN and the FATCOD scales) from English into Afaan Oromoo language, the standard forward–backward procedure was applied. Firstly, the translation was done by three authors (W.E., B.W., R.T.) who are nurse educators, clinically experienced and experts in research activities. Their native language is Afaan Oromoo. The English language is the medium of instruction for nurses in Ethiopia. Since the study aimed to use the questionnaire with nurses working inpatient units, the items were discussed with two nurses working in ICU and internal medicine ward. We revised the constructive comments accordingly. Then, a Ph.D. teacher of the English language at Wollega University translated the questionnaire back. Both versions were compared by W.E., B.W., R.T. for any discrepancies. Afterward, a pretest was conducted among 21 nurses. They were asked to read the questionnaire and make their comments on it. Each item was discussed for both scales. All items, except item number 16, “Demerol is not an effective analgesic in the control of chronic pain” were clear, and no major changes made. Seventeen nurses reported that it was difficult to understand “Demerol”. We replaced it with “Pethidine”.

### Data collection procedure and quality assurance

After we obtained ethical clearance from ethical review committee of Wollega University, Institute of Health Science, we made contact with each hospital’s medical director to grant permission with a copy of an approved ethical clearance letter to undertake the study. All selected hospitals’ medical directors readily accepted our request. Secondly, the head nurses were asked for their cooperation to give the permanent nurses staff list in their unit. To each hospital, their respective matron and two data collection facilitators were recruited. The packets of questionnaires were distributed to the 8 public hospitals by the research team. The nursing staff was given an oral explanation about the purpose of the study, the inclusion and exclusion criteria for the study, procedures of the study, the time it would take to complete the questionnaires (30–35 minutes), the confidentiality of data, a guarantee of voluntary and anonymous participation and that they could withdraw from the study at any time without fear or prejudice. All nurses who gave consent and volunteered were asked to fill in the questionnaire and return the completed questionnaire to the data facilitators.

### Data analysis

Data obtained were coded and entered into the computer using EPI data version 3.1 statistical packages. The Statistical Package for Social Sciences (SPSS) version 20.0 (IBM Corporation, Armonk, NY) used for data analysis. Categorical variables have computed as frequencies and percentages. Continuous variables (knowledge and attitude) are compiled as mean and standard deviation (SD). In this study, dependent variables (knowledge and attitude) were dichotomized based on the mean score. In the knowledge domain, a total score of greater than mean was considered as “adequate” knowledge, while score less-than-or-equal-to mean signified “inadequate” knowledge in the knowledge domain. Similarly, the attitude was positive if participants gained a score more than 50% of the total attitude score, while a total score < = 50% was considered a negative attitude towards end-of-life care. Binary logistic regression was employed to assess the association between dependents and independent variables. The significance level was set at 0.05.

### Ethical considerations

Before data collection, ethical clearance approval was obtained from ethical review committee of Wollega University, Institute of Health Science (reference number: WU/141, 666/RES1-26). Supportive official letter was also obtained from the respective hospitals to collect data from the participants. Participants were told the aim of the study and informed written consent was obtained from nurses. The confidentiality and anonymity of the questionnaires were guaranteed before the study started. Furthermore, they also were informed that they could withdraw from the study at any time.

## Results

### Demographic characteristics of nurses

This study was analyzed based on 372 nurses who participated in the study, for a response rate of 88%. The age of respondents ranges from 20 to 58 years, with a mean age of 29.23 ± 5.52. More than half (57.3%) of the participant nurses were male. Seventy-one percent (71%) of them had been working in primary and general hospitals. One in five respondents (19.9%) had palliative care service experience while only 16.4% of them had attended palliative care lectures/training. Besides, 129 (34.7%) had ever read articles or brochures about palliative care. Around 12% of the respondents received training or attended lecture regarding palliative care at nursing schools (Table 1).

**Table 1:** Demographic characteristics of nurses working in public hospitals in Wollega zones, 2019.

Variables	Frequency	Percentage
Level of hospital		
• Primary	70	18.8
• General	153	41.2
• Referral	98	26.3
• Specialized	51	13.7
Age in years (mean = 29, SD = 23 5.52)		
• 20–29	232	62.4
• 30–39	115	30.9
• > = 40	25	6.7
Gender		
• Male	213	57.3
• Female	159	42.7
Cumulative grade point average		
• <2.75	72	19.4
• 2.75–3.5	200	53.8
• >3.5	100	26.9
Nursing profession experience duration (years, mean = 6.23, SD = 4.2)		
• 1–5	196	52.7
• 6–10	127	34.1
• > = 11	49	13.2
Education level		
• Degree and above	303	81.5
• Diploma	69	18.5
Palliative care service experience		
• Yes	74	19.9
• No	298	80.1
Palliative care service experience in years (mean = 3.45, SD = 2.7)		
• 1–2	31	8.3
• 3–4	10	2.7
• > 4	17	4.6
• Missed	314	84.4
Had ever attended PC lectures/training		
• Yes	61	16.4
• No	311	83.6
Place where PC lectures/training attended		
• At university	31	8.4
• On job	17	4.5
• At college	13	3.5
Work unit		
• Medical/surgical	125	33.6
• Pediatrics/neonatal	91	24.5
• Maternal	47	12.6
• ICU/Emergency/ORT	66	17.7
• Others	43	11.6
Number of peoples received PC		
• 1–10	162	43.5
• >10	89	23.9
Read articles/brochures about PC		
• Yes	129	34.7
• No	243	65.3
Current work status		
• Director/matron/head	45	12.1
• Staff	327	87.9

### Nurses’ knowledge about palliative care

More than half (51.8%) of the study participants had scored above the mean cut off point (9.34, SD = 2.28). This study showed that only 0.5% of the participants had scores exceeding a cutoff score of 15, representing adequate knowledge about palliative care. More than half (51.6%) of the respondents had scored below 10 from 20 scales of PCQN items. The rate of correct answers ranged from 89% to 5.6%. The highest (69.4%) and lowest (10%) mean item score belonged to the theme management of pain and other symptoms, and psychosocial and spiritual care, respectively. Similarly, both the highest and lowest correct answers belonged to theme management of pain and other symptoms, item number 4 which said that adjuvant therapies are important in managing pain (89% answered correctly) and item number 3 that said the extent of the disease determines the method of pain treatment (5.6%), respectively (Table 2).

**Table 2:** Distribution of nurses’ knowledge about palliative care on the PCQN scale.

No-	PCQN Items	Correct, n (%)	Incorrect, n (%)	Don’t’ know, n (%)
Theme 1: Philosophy and principle of palliative care (1.93±1.0), 20.6%				
Q1	Palliative care is appropriate only in situations where there is evidence of a downhill trajectory or deterioration. (F)	258(69.4)	97 (26.1)	17(4.6)
Q9	The provision of palliative care requires emotional detachment. (F)	262(70.4)	89(23.9)	21(5.6)
Q12	The philosophy of palliative care is compatible with that of aggressive treatment. (T)	186 (50)	108(29)	78(21)
Q17	The accumulation of losses renders burnout inevitable for those who seek work in palliative care. (F)	186(50)	124 (33.3)	62(16.7)
Theme 2: Psychosocial and spiritual care (0.93±0.83), 10%				
Q5	It is crucial for family members to remain at the bedside until death occurs. (F)	72(19.4)	283(76.1)	17(4.6)
Q11	Men generally reconcile their grief more quickly than women. (F)	114(30.6)	228 (61.3)	30(8.1)
Q19	The loss of a distant or contentious relationship is easier to resolve than the loss of one that is close or intimate. (F)	161(43.3)	187 (50.3)	24(6.5)
Theme 3: Management of pain and other symptoms (6.48±1.8), 69.4%				
Q2	Morphine is the standard used to compare the analgesic effect of other opioids. (T)	261(70.2)	71(19.1)	40(10.8)
Q3	The extent of the disease determines the method of pain treatment.(F)	21(5.6)	333 (89.5)	18(4.8)
Q4	Adjuvant therapies are important in managing pain. (T)	331 (89)	24(6.5)	17(4.6)
Q6	During the last days of life, the drowsiness associated with electrolyte imbalance may decrease the need for sedation. (T)	259 (69.6)	83(22.3)	30(8.1)
Q7	Drug addiction is a major problem when morphine is used on a long-term basis for the management of pain. (F)	66(17.7)	282 (75.8)	24(6.5)
Q8	Individuals who are taking opioids should also follow a bowel regime. (T)	269 (72.3)	52(14)	51(13.7)
Q10	During the terminal stages of illness, drugs that can cause respiratory depression are appropriate for the treatment of severe dyspnea. (T)	162 (43.5)	177(47.6)	33 (8.9)
Q13	The use of placebos is appropriate in the treatment of some types of pain. (F)	137(36.8)	207(55.6)	28(7.5)
Q14	In high doses, codeine causes more nausea and vomiting than morphine. (T)	249 (66.9)	66(17.7)	57(15.3)
Q15	Suffering and physical pain are synonymous. (F)	187(50.3)	161(43.3)	24(6.5)
Q16	Pethidine is not an effective analgesic in the control of chronic pain. (T)	92(24.7)	173(46.5)	107(28.8)
Q18	Manifestations of chronic pain are different from those of acute pain. (T)	297(79.8)	62(16.7)	13 (3.5)
Q20	The pain threshold is lowered by anxiety or fatigue. (T)	81(21.8)	270(72.6)	21(5.6)
	PCQN overall score (Mean = 9.34, SD = 2.28)	Frequency (%)		
	• 0–5	17(4.6)		
	• 6–10	253 (68)		
	• 11–15	100 (26.9)		
	• 15+	2 (0.5)		

### Nurses’ attitude toward end-of-life care on the FATCOD scale

The study result showed that the total FATCOD score ranges 61–100 (mean = 79.58, median = 80, SD = 6.33), but only 19 (5.1%) had a score greater than75% for FATCOD scales. The respondents predominantly hold a positive and supportive attitude towards ends of life care. The data analysis of the nurses’ attitudes towards end-of-life care showed nurses the degree of nurses’ opinion concerning certain statements (Table 3). A majority (78.7%) of the subjects supported the thought that the involvement of non-family caregivers should continue as the patient nears death. Most nurses also believed that non-family caregivers can help patients prepare for death (92.8), families should maintain as normal an environment as possible for their dying member (93%) and giving care to the dying person is a worthwhile experience (89.9%) (Table 3).

**Table 3:** Frequency distribution of nurses’ attitude towards end of life care on FATCOD scale.

No-	FATCOD Items	Mean (SD)	SD, n (%)	DA, n (%)	Un, n (%)	A, n(%)	SA, n (%)
1.	Palliative care is given only for dying patient	2.78(1.30)	53(14.2)	107(28.8)	66(17.7)	93(25)	53(14.2)
2.	As a patient nears death; the nurse should withdraw from his/her involvement	3.97(1.16)	146(39.2)	147(39.2)	21(5.6)	37(9.9)	21(5.6)
3.	Giving nursing care to a dying person is a worthwhile learning experience.	4.24(0.75)	3(0.8)	10(2.7)	24(6.4)	192(51.6)	143(38.4)
4.	It is beneficial for the dying person to verbalize his/her feelings	4.33(0.73)	3(0.8)	6(1.6)	23(6.2)	173(46.5)	167(44.9)
5.	Family members who stay close to a dying person often interfere with a professionals' job with the patient.	3.33(.24)	34(9.1)	77(20.7)	56(15.1)	140(37.6)	65(17.5)
6.	The length of time required to give nursing care to a dying person would frustrate me.	3.09(1.23)	46(12.4)	123(33.1)	64(17.2)	97(26.1)	42(11.3)
7.	Families should be concerned about helping their dying member make the	4.20(0.91)	4(1.1)	25(6.7)	27(7.3)	152(40.9)	164(44.1)
8.	Family should maintain as normal an environment as possible for their dying member	4.47(0.67)	1(0.3)	4(1.1)	21(5.6)	137(36.8)	209(56.2)
9.	The nurse should not be the one to talk about death with the dying person.	1.89(1.11)	17(4.6)	27(7.3)	30(8.1)	122(32.8)	176(47.3)
10.	The family should be involved in the physical care of the dying person.	4.12(0.91)	5(1.3)	21(5.6)	43(11.6)	157(42.2)	146(39.2)
11.	It is difficult to form a close relationship with the family of a dying member	3.04(1.29)	53(14.2)	107(28.8)	66(17.7)	93(25)	53(14.2)
12.	There are times when death is welcomed by the dying person	3.74(1.08)	11(3)	48(12.9)	66(17.7)	146(39.2)	101(27.2)
13.	Nursing care for the patient's family should continue throughout the period of grief and bereavement.	3.44(1.31)	26(7)	90(24.2)	54(14.5)	96(25.8)	106(28.5)
14.	The dying person and his/her family should be the in-charge decision makers	3.90(1.02)	8(2.2)	40(10.8)	46(12.4)	164(44.1)	114(30.6)
15.	Addiction to pain relieving medication should not be a nursing concern when dealing with a dying person	2.81(1.33)	71(19.1)	108(29)	60(16.1)	84(22.6)	49(13.2)
16.	Nursing care should extend to the family of the dying person	4.07(0.97)	8(2.2)	31(8.3)	21(5.6)	176(47.3)	136(36.6)
17.	I think it is best to change the subject to something cheerful when a patient asks, “Nurse am I dying?	2.12(1.15)	22(5.9)	37(9.9)	30(8.1)	158(42.5)	127(33.6)
18.	I am afraid to become friends with chronically sick and dying patients	3.29(1.21)	54(14.5)	140(37.6)	76(20.4)	65(16.9)	39(10.5)
19.	I would be uncomfortable if I entered the room of a terminally ill person and found him/her crying	2.62(1.19)	28(7.5)	75(20.2)	58(15.6)	150(40.3)	61(16.4)
20.	I would be uncomfortable talking about impending death with the dying Person	2.05(1.01)	11(3)	32(8.6)	39(10.5)	172(46.2)	118(31.7)
21.	It is possible for nurses to help patients prepare for death	1.56(0.76)	3(0.8)	10(2.7)	14(3.8)	139(37.4)	206(55.4)
22.	Death is not the worst thing that can happen to a person	3.00(1.37)	67(18)	88(23.7)	54(14.5)	102(27.4)	61(16.4)
23.	I would feel like running away when the person actually died	3.82(1.31)	145(39)	128(34.4)	23(6.2)	39(10.5)	37(9.9)
24.	I would not want to be assigned to care for a dying person	3.66(1.25)	109(29.3)	135(36.3)	51(13.7)	45(12.1)	32(8.6)
FATCOD cut-off overall score (mean = 79.58, SD = 6.33)							
• Score >79.58					189(51.8)		
• Score < = 79.58					183(49.2)		

### Factors associated with nurses’ knowledge about PC and attitude toward end-of-life care

The findings showed that only nurses’ who had PC service experience and attended lectures/training had a significant association with their mean total knowledge score. Having PC service experience [AOR 1.94 CI (1.10–3.42), p = 0.02] and had ever attended training/lecture on PC [AOR = 1.87 CI (1.01–3.46), p = 0.04] positively affected nurses’ knowledge about PC. However, the level of hospitals, respondents’ age, gender, the level of education, nursing work experience, clinical work units, place of training and reading articles/brochures about PC did not show a significant association.

It also indicated nurses’ attitude towards end-of-life care is significantly associated with the nurses who had palliative care service experience, nurses who read resources about PC (articles, brochures) and a number of people to whom nurses had provided palliative care. Nurses who had PC service experience were 0.4 times less likely showed a positive attitude than nurses who had no PC service experience towards end of life care [AOR = 0.41, CI(0.21–0.79), p = 0.008]. Meanwhile, nurses who read articles/brochures about PC were 1.94 times more likely displayed a positive attitude than nurses who did not read articles/brochures about palliative care [AOR = 1.94, CI (1.11–3.39), p = 0.01]. Nurses who had provided care for a smaller number of terminally ill patients were 1.74 times more likely [AOR = 1.74, CI (1.01–2.97), p = 0.04] showed a positive attitude towards end-of-life care (Table 4).

**Table 4:** Factors associated with nurses’ knowledge about PC and attitude towards–end-of-life care.

Variables			CO, 95% CI	AOR, 95% CI
Knowledge of nurses about PC	**Adequate, N (%)**	**Inadequate, N (%)**		
PC service experience				
• Yes	48(12.9)	26 (7)	2.32 (1.36–3.94)	1.94(1.10–3.42)*
• No	132 (35.5)	166 (44.6)	1.00	1.00
Had ever attended PC training/lecture				
• Yes	40(10.8)	21(5.6)	2.34(1.31–4.12)	1.87(1.01–3.46)*
• No	140(37.6)	171(46)	1.00	1.00
Read resources about PC (articles, brochures,)				
• Yes	69 (18.5)	60(16.1)	1.36 (1.08–2.09)	1.03(0.65–1.65)
• No	111(29.8)	132(35.5)	1.00	1.00
Attitude toward end-of-life care	**Positive**	**Negative**		
PC service experience				
• Yes	30(8.1)	44 (11.8)	0.63(0.38–1.06)	0.41(0.21–0.79)*
• No	154 (41.4)	144 (38.7)	1.00	1.00
Read resources about PC (articles, brochures,)				
• Yes	76 (20.4)	53(14.2)	1.75(1.16–2.76)	1.94(1.11–3.39)*
• No	108 (29)	132 (36.3)	1.00	1.00
Number of terminally ill peoples cared by nurses				
• 1–10	94(37.5)	68(27.1)	1.78 (1.05–2.98)	1.74(1.01–2.97)*
• >10	39(15.5)	50 (19.9)	1.00	1.00

## Discussion

The present study assessed the level of nurses’ knowledge about palliative care and attitudes towards ends of life care and associated nurses’ demographic variables in public hospitals in Wollega Zones. Nurses’ palliative care mean total knowledge score (9.34) appears to be deficient in this study. This finding is higher compared to the previous studies undertaken in Saudi Arabia (2009), Southeast Iran (2014), and Iran (2019) [20–22]. Meanwhile, our study result is lower matched to researches reported from Canada (2009), in the USA [2009], Australia (2008) and Ireland (2016) [17, 30–32]. The study results might vary due to differences in the curriculum (presence, absence or poor quality of palliative care courses in nursing education), limitation in training/refreshing staff nurses about palliative care and absence of resources used for reading such as internet connections, updated guidelines, library and articles/brochures.

The finding highlighted a gap in nurses’ knowledge about palliative care. However, most of them had higher mean knowledge about pain management and other symptoms (mean = 6.48). This is in line with Iranian nurses and Greece nursing students [21, 33]. The reason for higher knowledge score about pain management and other symptoms may be due to hospital nurses most frequently manage give care for chronically ill patients who require pain killers and perform it daily. It was also suggested that continuing professional development activities and active clinical practices as the possible reasons for higher mean knowledge scores on pain management and other symptoms.

Our findings also pointed out the nurses’ major key misconceptions. The first highest number of incorrect responses on the PCQN was for question number 3: ‘the extent of the disease determines the method of pain treatment (94.4%)’. This is similar to previous studies reported in Addis Ababa (85%) and Iran (83.4%) [19, 21]. This warrants that nurses should determine the right drug, dose, route and frequency of medication to treat pain. The 2^nd^ highest number of incorrect responses on the PCQN was for question 7: ‘drug addiction is a major problem when morphine is used on a long-term basis for the management of pain (82.3%)’. The reason could be due to either the participants did not assess their patient progress after morphine administration for addiction or opioid drugs such as morphine are not commonly available and given at primary and general hospitals in our country. Thirdly, the incorrectly responded was item number 5; ‘it is crucial for family members to remain at the bedside until death occurs (80.6%)’. This is in harmony with Iranian nurses’ response (81.1%) [21]. Cultural/religious factors shape society's expectations and the fundamental role of the family in the care of the dying patient [23]. In our country (Ethiopia), family members of dying patient are respected by nurses when they obtain psychological, spiritual and cultural support that help relieve their stress and avoid complex grief.

The mean score of the FATCOD scale was 79.58, more than half (51.8%) held a less favorable attitude towards caring for the dying. It is higher than the results found among nurses in Malaysia hospitals (60.19) [24]. This is lower than the findings in Addis Ababa (76%) and Iran (85.3%) [19, 21]. The difference of mean scores may be due to the variation of beliefs and cultures among nursing staffs across regions which need to be investigated.

In the present study, nurses who had PC service experience were (2.47 times) and who had ever attended PC lectures/ training (2.35 times) more knowledgeable than nurses who do not have PC service experience and had not ever attended lectures/training on palliative care. The former factor is strongly supported by the study finding undertaken in Addis Ababa [19]. These show us that the more nurses practice palliative care the better knowledge they acquire about it. This could be supported by the presence of library, electronic materials used for reading and hospitals provides training about palliative care for nurses could have a higher opportunity for improving their knowledge.

However, the level of hospital, age, sex, educational qualification, years of experience in nursing profession respondents, number of terminally ill patients nurse gave care and duration of training had no significant association with nurses’ knowledge score. This is supported by the study result in Iran [21]. However, studies displayed that as nurses’ years’ work of experience and level of education were significantly associated with nurses’ better knowledge score [21, 22]. In contrast, one study reported that nurses’ knowledge did not correlate with the training of palliative care [23].

In this study, nurses’ who had palliative care service experience (0.4 times), who read articles (1.94 times) and fewer terminally ill patients they had ever given care (1.74 times) showed a more favorable attitude toward end-of-life care. However, the study done in Western Australia displayed that nurses who received training about palliative care demonstrates a more positive attitude than nurses who did not receive training about PC.

Our analysis displayed level of hospitals, education qualification and clinical working units has no significant association with the nurses’ attitude toward end-of-life care. However, a higher level of education, working in a medical ward, and nurses received training on PC showed a more favorable attitude towards PC [17, 19, 22]. The experience of nurses and attitude toward end-of-life care is incongruent to each other in this study which might be affected by nurses’ belief (religion and culture), their compassion and experience adapted from family members. As education or training can bring behavioral change, nurses who read articles about palliative can bring a favorable attitude end-of-life care is suggested.

Nurses’ attitude toward end-of-life care had no significant association with their length of duration in the nursing profession experience. In contrast, it is argued that nurses’ attitude toward death and dying are positively affected by experience [34]. In future study, it is better to investigate the association between nurses’ religion/spirit and their attitude toward end-of-life care.

### Conclusion

The study generated evidence that indicates inadequate knowledge about palliative care and a less favorable attitude towards end-of-life care among nurses working in public hospitals in Wollega zones. It also indicated nurses working in palliative care units help improving nurses’ knowledge about PC. Our findings also suggested reading resources about PC and giving care for more terminally ill patients positively improve nurses’ attitude towards end-of-life care. These suggest that the core structured part of palliative care in nursing.

### Strength and limitation of the study

The study was multicenter and analyzed from a large size, which may enhance the generalization of these findings. Furthermore, we utilized internationally standardized tools. Similarly, the study tool was translated into a local language to minimize cultural and language discrepancy despite its impact on the validity of the questionnaire. The pretest was undertaken among 21 nurses on duty. However, the cross-sectional study design allows determining associations but not causal relationships in the analysis of potential predictors of nurses’ knowledge and attitude. Moreover, a self-report questionnaire can result in a low response rate and has a potenstial bias (confounding by misremembering and overstating a palliative care experience).

## Supporting information

S1 File (DOCX)

S2 File (DOCX)

S3 File (SAV)

## Abbreviations

FATCOD: Frommelt Attitudes Toward Care of the Dying Scale
NCDs: Non-Communicable Diseases
PCQN: Palliative Care Quiz For Nursing
PC: Palliative Care
PHS: Public Health Strategy
SPSS: Statistical package for social sciences
WHO: World Health Organization
