# Supplementary material for: Nurses’ knowledge about palliative care and attitude towards end- of-life care in public hospitals in Wollega zones: A multicenter cross-sectional study
Source: PLoS One. 2020 Oct 7;15(10):e0238357. doi: 10.1371/journal.pone.0238357 (PMC7540839; doi:10.1371/journal.pone.0238357)
Supplement: S1 File — (DOCX) [file pone.0238357.s001.docx]

**MATA DUREE QORANNOO: NURSES’ KNWLEDGE & ATTITUDE ABOUT PALLIATIVE CARE IN PUBLIC HOSPITALS IN WOLLEGA ZONES, 2019: Cross sectional study design yoo ta’u gaaffileen kun Afaan kan qophaa’nii fi kutaalee sadii qabu.**

| **Kutaa 1ffaa: Gaaffileen armaan gadii odeeffannoo walii gala ogeeyyii narsiingii ilaallata. Deebii keessan haala gaafatamtaniin akka nuuf deebiftan kabajaan isin gaafanna.** | | | | | | |  |  |
| --- | --- | --- | --- | --- | --- | --- | --- | --- |
| S.NO | | Questions/Gaaffilee | Responses/Deebii/ itti mari ykn iddoo duuwwaarra guuti | | | |  |  |
|  | | Hospitaalli ati keessaa hojjettu sadarkaansaa | 1. Kan jalqabaa 2. Riffeeraalii 3. Walii galaa 4. Speeshaalaayzidii | | | |  |  |
| 1. 2. | | Umriinkee waggaa meeqa ta’a? | Waggaa________________ | | | |  |  |
| 1. 3 | | Saallikee kami? | 1. Dhiira 2. Dhalaa | | | |  |  |
|  | | Qabxiin Waliigalaaa inni yeroo dhiyootti ittiin eebbifamtee meeqa ture? | 1. <2.75 gadi 2. 2.75- 3.5 3. >3.5 | | | |  |  |
|  | | Muuxannoo ogummaa narsiingiitiin waggaa meeqa tajaajilte? | Waggaa________________ | | | |  |  |
|  | | Sadarkaaan Barnootakee ammaa itti muudamtee jirtu kami? | 1. Degree 2. Diploma 3. Msc | | | |  |  |
|  | | Muuxannoo kutaa tajaajila kunuunsa paleetiivii (palliative care) qabdaa? | 1. Eeyee 2. Lakki | | | |  |  |
|  | | Gaaffii 7ffaa’f **eeyyee** yoo jette, muuxannoon ati kunuunsa paleetiivii ilaaclchisee qabdu waggaa meeqa ta’a? | Waggaa_________ | | | |  |  |
|  | | Paleetivii ilaalchisee leenjii fudhatteee beektaa? | 1. Eeyyee 2. Lakki | | | |  |  |
|  | | Yoo, gaaffiin lakk 10’f ‘eeyyee’jette, leenjii eessatti fudhatte? | 1. Barnoota yuunivarsiitiirratti 2. Hojiirra yeroon jiru 3. Barnoota dippiloomaarratti 4. Kanbiroo____________________ | | | |  |  |
|  | | Yeroo ammaa kutaan ati tajaajila ogummaa narsiingii kennaa jirtu isa kami? | 1. Medical/ Surgical 2. Pediatrics/Neonatal 3. Gynecology/obstetrics 4. Emergency/ICU/ORT 5. Others | | | |  |  |
|  | | Waggaa darbe kana keessatti, dhukkubsattoota hagamtu tajaajila palliative care sirraa argate? | Lakkoofsaan _______________________ | | | |  |  |
|  | | Waa’ee Paleetiivii ilalachisee barruulee ykn qorannoolee bahan ni dubbistaa? | 1. Eeyyee 2. Lakki | | | |  |  |
| Kutaa 2ffaa: Gaaffilee armaan gadii dhugaa, soba ykn hin beeku jechun deebisaa. Deebii keessan fuula dura gaaffiitti qixa filannoo deebii isiniif laataman jalaan gadi mallattoo right mark (✓,)kaayaa | | | | | | | | |
| An instrument to measure nurses' knowledge in palliative care (Gaaffilee) | | | | | Dhugaa | Soba | Hinbeeku | |
|  | | Tajaajilli dhibee hin fayyamnee (palliative care) yeroo haalli fayyaa dhukkubsaataa baayyee yaaddessaa ta’e qofa kennamuu qaba | | |  |  |  | |
|  | | Qorichi ‘morphine’ qorichoota dhukkubbii hir’isaan biroo kanneen akka opiods itti madaaluuf akka waltawaa (standard) tti fayyadudha. | | |  |  |  | |
|  | | Sadarkaan dhukkubbii adeemsa dhukkubbiin itti yaalamu ni murteeessa. | | |  |  |  | |
|  | | Dhukkubbii wal’aanuuf, wal’aansi dabalataa(adjuvant therapy) barbaachisaadha | | |  |  |  | |
|  | | Haga dhukkubsataan du’utti, maatiin isaa/ishee biraa dhabamuu hin qaban | | |  |  |  | |
|  | | Yeroo lubbuun bahuuf jedhutti, sababa ‘electrolyte imbalance’ tiin dhukkubsataan waan ofwallallaaluuf, qorichi hadoochu kennuufiin hagas mara barbaachisaa miti | | |  |  |  | |
|  | | Moorfinii yeroo dheeraaf dhukkubbii hambisuuf fayyadamuun rakkoo suusii qorichaaf ni saaxiala | | |  |  |  | |
|  | | Namni qorichoota Opiods/ dhukkubbii ajjeesan/ fudhatu sirna bobbaasaanii hordofamuu qaba | | |  |  |  | |
|  | | Tajaajila dhukkuba hin fayyamnee (palliative care) kennuuf, miira keessa ofgalchuun barbaachisaa miti | | |  |  |  | |
|  | | Qorichoonni sirna hargansuu dadhabsiisan sadarkaa dhukkubbii isa dhumaa irratti kennuun rakkoo hargansuu hamaa yaaluuf ni barbaachisu. | | |  |  |  | |
|  | | Dhiirotni dubartoota caalaa dafanii gadda isaan mudate keessaa ni bahu | | |  |  |  | |
|  | | Filoosoofiin Tajaajila dhukkuba hin fayyamnee (palliative care), yaala murteessaa fi barbaachisaa (aggressive treatment) wajjin walfakkaata | | |  |  |  | |
|  | | Dhukkubbii tokko tokkoof Qoricha fakkeessaa (placebo)tti fayyadamuun barbaachisaadha | | |  |  |  | |
|  | | Yoo qorichi codaine jedhamu baayyinaan (high dose) fudhatame, morphine caalaa rakkoo oldeebisuu (nausea and vomiting) fiduu danda’a | | |  |  |  | |
|  | | Dararamuu fi dhukkubbiin qaamaa wal fakkaataadha | | |  |  |  | |
|  | | Qorichi ‘demerol’ jedhamu dhukkubbii nama irra ture (chronic pain) wal’aanuuf bu’aa qabeessa miti. | | |  |  |  | |
|  | | Dhukkubasataa baay’inaan dhabuun gadda dhorkamuu hin dandeenye /kan qabbana hinqabneef/ warra Palliative care keessaa hojjetaniif saaxila. | | |  |  |  | |
|  | | Mallattooleen dhukkuba nama irra turanii/qancraa (chronic pain) mallattoolee dhukkubbii hatattamaa (acute pain) irraa garaagara. | | |  |  |  | |
|  | | Gaddi nama walitti dhufeenya hin qabne dhabuu, kan nama baay’ee walitti dhiyaatan dhabuu irra salphaadha. | | |  |  |  | |
|  | | Dhiphinni ykn dadhabbiin hamma dhukkubbii ni xiqqeessa. | | |  |  |  | |

**Section III: Gaaffilee Ilaalcha ogeessi narsii Pallitaive care tiif qabu/bdu qorata**

**Attitude testing items**

| Items testing items  Gaaffiilee ilaalcha ogeessi narsii “palliative care” tiif qabu qoratan | | Strongly agree | Agree | Neither agree nor disagree | Disagree | Strongly disagree |
| --- | --- | --- | --- | --- | --- | --- |
|  | Kunuunsi Paaleetiivi dhukkubsattoota fayyuu hin dandeenye qofaaf kennama |  |  |  |  |  |
|  | Yeroo dhukkubsataan gara du’uu jala gahu, narsiin kunuunsa gochuu qabu dhaabuu qaba |  |  |  |  |  |
|  | Dhukkubsataa dhibee qancaraa qabuuf kunuunsa narsiingii kennuun muuxannoo baratamuu qabu argachuuf ni fayyada. |  |  |  |  |  |
|  | Namoonni dhukkuba yeroo dheeraa qaban wanta isaanitti dhagahame himachuun isaan fayyada |  |  |  |  |  |
|  | Miseensi Maatiin dhukkubsataa gara lubbuun darbuutti dhiyaate waliin jiru yeroo baay’ee hojii ogeessi dhukkubsatichaaf godhu gidduu ni galu. |  |  |  |  |  |
|  | Dheerinni yeroo kunuunsaa narsiin nama du’uuf ka’eef kennu na sodaachisa/yaaddessa. |  |  |  |  |  |
|  | Nama du’uu jala gaheef haftee yeroo jireenya isaa gaarii gochuutti maatiinsaa xiyyeeffachuu qabu. |  |  |  |  |  |
|  | Maatiin dhukkubsatichaa haalota inni keessa jiraatu amma danda’anitti gaarii fi mijataa godhanii eeguu qabu |  |  |  |  |  |
|  | Narsiin nama duuuf ka’etti waa’ee du’aa haasa’uun irraa hin eegamu. |  |  |  |  |  |
|  | Maatiin kunuunsa qaama nama du’uuf ka’ee irratti hirmaachuu qabu. |  |  |  |  |  |
|  | Hariiroo walitti dhiheenya maatii nama lubbuun darbuuf jedhu waliin uumun ulfaataadha |  |  |  |  |  |
|  | Yeroon itti namni lubbuun darbuu gahe du’a barbaadu ni jira |  |  |  |  |  |
|  | Kunuunsi narsiin maatii dhukkubsataan jalaa du’eef kennu amma yeroo isaan gadda dabarsanitti itti fufuu qaba |  |  |  |  |  |
|  | Nama lubbuun darbuuf jedhuu fi maatiin isaa wanta kamiifuu warra murtee kennan ta’uu qabu |  |  |  |  |  |
|  | Narsiin yerooma nama fayyuu hin dandeenye kunuunsutti, waa’een araada dawaa miira dhukkubbii ittisuun qabamuun dhukkubsatichaa xiyyeeffatamuu hin qabu |  |  |  |  |  |
|  | Kunuunsi narsiin kennuu dhukkubsaticha irra darbee maatiisaatifis ta’uu qaba |  |  |  |  |  |
|  | Yeroo dhukkubsataan Narsiidhaan ‘’ani nan du’aa’’? jedhee gaafatu, yaadicha gara waanta gaaritti jijjiirun gaariidha jedheen yaada |  |  |  |  |  |
|  | Namoota dhukkuba yeroo dheeraa dhukkubsatanii fi gara du’atti dhihaatan waliin hiriyoomuu nan sodaadha |  |  |  |  |  |
|  | Kutaa nama du’uuf ka’ee seenee isaa boohu/isheetii boossu yoon arge natti hin tolu. |  |  |  |  |  |
|  | Nama du’uuf ka’etti waae’ee du’aa haasa’uun natti hin tolu |  |  |  |  |  |
|  | Dhukkubsataa du’a jala ga’eef narsiin gargaaruu qaba/qabdi |  |  |  |  |  |
|  | Duuti wanta hundumarra hamaa namatti dhufu miti |  |  |  |  |  |
|  | Dhukkubsataan yeroo du’u fiigichaan kutaa sana gadhiisuun qaba. |  |  |  |  |  |
|  | Nama du’uuf jiru akkan kunuunsuuf ramadamuu hin fedhu |  |  |  |  |  |

Galatoomaa!
