# Supplementary material for: Nurses’ knowledge about palliative care and attitude towards end- of-life care in public hospitals in Wollega zones: A multicenter cross-sectional study
Source: PLoS One. 2020 Oct 7;15(10):e0238357. doi: 10.1371/journal.pone.0238357 (PMC7540839; doi:10.1371/journal.pone.0238357)
Supplement: S2 File — (DOCX) [file pone.0238357.s002.docx]

**A QUESTIONNARE TO ASSESS NURSES’ KNOWLDEGE ABOUT PALLIATIVE CARE AND ATTITUDE TOWARDS END OF LIFE CARE IN PUBLIC HOSPITALS IN WOLLEGA ZONES**

| **Section I: Demographic characteristics of participant** | | | | | | | | | | | |
| --- | --- | --- | --- | --- | --- | --- | --- | --- | --- | --- | --- |
| SNO- | | Questions | | Responses (Encircle to your correct answer ) | | | | | | | |
|  | | What is your hospital level? | | 1. Primary 2. General 3. Referral 4. Specialized | | | | | | | |
|  | | How old is your age? | | ________________years | | | | | | | |
|  | | What is your gender? | | 1. Male 2. Female | | | | | | | |
|  | | Which category indicates your total cumulative grade point average? | | 1. <2.75 2. 2.75-3.5 3. >3.5 | | | | | | | |
|  | | How long is your year of experience in professional nursing? | | ________________years | | | | | | | |
|  | | What is your current level of education? | | 1. Degree 2. Diploma 3. Masters | | | | | | | |
|  | | Do you have an experience of working in palliative care unit? | | 1. Yes 2. No | | | | | | | |
|  | | If yes to question number 7, how long your experience? | | __________________years | | | | | | | |
|  | | Have you ever attended or received training about palliative care? | | 1. Yes 2. No | | | | | | | |
|  | | If yes to question number 10, where did you attend training about palliative care? | | 1. At University education 2. On Job training 3. At Diploma education 4. Others | | | | | | | |
|  | | Where is your current clinical unit you practiced palliative care? | | 1. Medical /Surgical 2. Pediatrics/ Neonatal 3. Maternal units 4. ICU/Emergency/ORT 5. Others | | | | | | | |
|  | | What is the number of terminally ill patients who were cared for in the past year? | | ________________ | | | | | | | |
|  | | Have you ever read articles/brochures/books about Palliative care? | | 1. Yes 2. No | | | | | | | |
| Answer the following questions saying true or false. Tick under column “**True”** if it is right, under the column “**False”** if it is right and under “**I Don’t know”** if you do not know the answer put the mark under column. | | | | | | | | | | | |
| An instrument to measure nurses' knowledge in palliative care | | | | | | True | | False | | I don’t know | |
|  | Palliative care is appropriate only in situations where there is evidence of a downhill trajectory or deterioration | | | | |  | |  | |  | |
|  | Morphine is the standard used to compare the analgesic effect of other opioids | | | | |  | |  | |  | |
|  | The extent of the disease determines the method of pain treatment | | | | |  | |  | |  | |
|  | Adjuvant therapies are important in managing pain | | | | |  | |  | |  | |
|  | It is crucial for family members to remain at the bedside until death occurs | | | | |  | |  | |  | |
|  | During the last days of life, the drowsiness associated with electrolyte imbalance may decrease the need for sedation | | | | |  | |  | |  | |
|  | Drug addiction is a major problem when morphine is used on a long-term basis for the management of pain | | | | |  | |  | |  | |
|  | Individuals who are taking opioids should also follow a bowel regime | | | | |  | |  | |  | |
|  | The provision of palliative care requires emotional detachment | | | | |  | |  | |  | |
|  | During the terminal stages of an illness, drugs that can cause respiratory depression are appropriate for the treatment of severe dyspnea | | | | |  | |  | |  | |
|  | Men generally reconcile their grief more quickly than women | | | | |  | |  | |  | |
|  | The philosophy of palliative care is compatible with that of aggressive treatment | | | | |  | |  | |  | |
|  | The use of placebos is appropriate in the treatment of some types of pain | | | | |  | |  | |  | |
|  | In high doses, codeine causes more nausea and vomiting than morphine | | | | |  | |  | |  | |
|  | Suffering and physical pain are synonymous | | | | |  | |  | |  | |
|  | Demerol is not an effective analgesic in the control of chronic pain | | | | |  | |  | |  | |
|  | The accumulation of losses renders burnout inevitable for those who seek work in palliative care | | | | |  | |  | |  | |
|  | Manifestations of chronic pain are different from those of acute pain | | | | |  | |  | |  | |
|  | The loss of a distant or contentious relationship is easier to resolve than the loss of one that is close or  intimate | | | | |  | |  | |  | |
|  | The pain threshold is lowered by anxiety or fatigue | | | | |  | |  | |  | |
| **Section III: Please, try to answer the following questions choosing Strongly agree, agree, unsure, disagree or strongly disagree. Put tick mark under your response. (SA=strongly agree, AG=agree, UN=unsure, DA=disagree and SDA= strongly disagree )** | | | | | | | | | | | |
| Items | | | SA | | AG | | UNS | | DA | | SDA |
|  | Palliative care is given only for dying patient. | |  | |  | |  | |  | |  |
|  | As a patient nears death; the nurse should withdraw from his/her involvement | |  | |  | |  | |  | |  |
|  | Giving nursing care to the chronically sick patient is a worthwhile learning experience. | |  | |  | |  | |  | |  |
|  | It is beneficial for the chronically sick person to verbalize his/her feelings | |  | |  | |  | |  | |  |
|  | Family members who stay close to a dying person often interfere with a professionals' job with the patient. | |  | |  | |  | |  | |  |
|  | The length of time required to give nursing care to a dying person would frustrate me. | |  | |  | |  | |  | |  |
|  | Families should be concerned about helping their dying member to make the best of his/her remaining life. | |  | |  | |  | |  | |  |
|  | Family should maintain as normal an environment as possible for their dying member | |  | |  | |  | |  | |  |
|  | The nurse should not be the one to talk about death with the dying person. | |  | |  | |  | |  | |  |
|  | The family should be involved in the physical care of the dying person. | |  | |  | |  | |  | |  |
|  | It is difficult to form a close relationship with the family of a dying member. | |  | |  | |  | |  | |  |
|  | There are times when death is welcomed by the dying person | |  | |  | |  | |  | |  |
|  | Nursing care for the patient's family should continue throughout the period of grief and bereavement. | |  | |  | |  | |  | |  |
|  | The dying person and his/her family should be the in-charge decision makers. | |  | |  | |  | |  | |  |
|  | Addiction to pain relieving medication should not be a nursing concern when dealing with a dying person. | |  | |  | |  | |  | |  |
|  | Nursing care should extend to the family of the dying person | |  | |  | |  | |  | |  |
|  | When a patient asks, “Nurse am I dying?'I think it is best to change the subject to something cheerful. | |  | |  | |  | |  | |  |
|  | I am afraid to become friends with chronically sick and dying patients | |  | |  | |  | |  | |  |
|  | I would be uncomfortable if I entered the room of a terminally ill person and found him/her crying | |  | |  | |  | |  | |  |
|  | I would be uncomfortable talking about impending death with the dying Person | |  | |  | |  | |  | |  |
|  | It is possible for nurses to help patients prepare for d | |  | |  | |  | |  | |  |
|  | Death is not the worst thing that can happen to a person | |  | |  | |  | |  | |  |
|  | I would feel like running away when the person actually died | |  | |  | |  | |  | |  |
|  | I would not want to be assigned to care for a dying person | |  | |  | |  | |  | |  |
